# Medullary unidentified bright objects in Neurofibromatosis type 1: a case series

**DOI:** 10.1186/s12887-018-1067-1

**Published:** 2018-02-28

**Authors:** Alessandra D’Amico, Federica Mazio, Lorenzo Ugga, Renato Cuocolo, Mario Cirillo, Claudia Santoro, Silverio Perrotta, Daniela Melis, Arturo Brunetti

**Affiliations:** 1 Department of Advanced Biomedical Sciences, “Federico II” University, via Sergio Pansini 5, 80100 Naples, Italy; 2 Department of Medical, Surgical, Neurological, Metabolic and Aging Sciences, “Seconda Università degli Studi di Napoli” University, via Costantinopoli 104, 80100 Naples, Italy; 3 Regional Referral Center for Neurofibromatosis, Department of Woman, Child, General and Specialistic Surgery, “Seconda Università degli Studi di Napoli” University, via Costantinopoli 104, 80100 Naples, Italy; 4 Department of Translational Medical Sciences, “Federico II” University, via Sergio Pansini 5, 80100 Naples, Italy; 5 Naples, Italy

**Keywords:** Neurofibromatosis I, Unidentified bright objects, Spine, Magnetic resonance imaging, T2-hyperintense lesions

## Abstract

**Background:**

In Neurofibromatosis type 1, cerebral Unidentified Bright Objects are a well-known benign entity that has been extensively reported in the literature. In our case series, we wish to focus on a further possible location of such lesions, the spinal cord, which we have defined as medullary Unidentified Bright Objects. These have been, to our knowledge, scarcely described in previous works.

**Case presentation:**

We report the cases of 7 patients with medullary Unidentified Bright Objects in Neurofibromatosis type 1 that we have followed for up to 9 years in our Regional Referral Center for Neurofibromatosis. In all of our patients, these lesions were completely asymptomatic and reported on Magnetic Resonance exams the patients underwent for other clinical indications.

**Conclusions:**

The aim of our work is to increase awareness of the possibility of medullary Unidentified Bright Objects in Neurofibromatosis type 1 patients, which can simulate neoplastic lesions, suggesting a more conservative approach in these cases.

## Background

Neurofibromatosis type 1 (NF1) is a rare (prevalence of 1 in 2700 newborns) autosomal dominant genetic disorder [1]. It is diagnosed on the basis of clinical criteria such as the presence of cutaneous spots (café-au-lait), freckling in the axillary or inguinal regions, neurofibromas (plexiform or otherwise), Lisch nodules, optic pilocityc astrocytoma, sphenoid wing hypoplasia or aplasia, long bone dysplasia and/ or pseudoarthrosis, and a positive family history (NIH criteria). On Magnetic Resonance (MR), NF1 presents a wide spectrum of alterations which involve both the white and gray matter in the whole CNS [2–5]. Spinal neoplasms in NF1 patients can be both extramedullary (neurofibromas and malignant peripheral nerve sheath tumors) and intramedullary (astrocytomas, ependymomas and gangliogliomas). We report seven cases of NF1, followed at our referral center, who shared intramedullary alterations characterized by multiple foci of high signal in T2WI similar to unidentified bright objects (UBOs) normally reported in the brain of NF1 patients [6, 7]. Therefore, medullary UBOs (mUBOs), although a rare finding, must be considered in the MRI features of patients with NF1.

## Case presentation

### Case 1

**Fig. 1:**
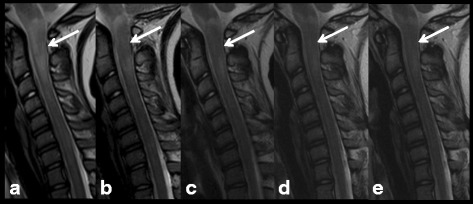
Patient number 1. Sagittal Turbo Spin-Echo T2-w sequences: a large intramedullary hyperintense lesion (C1-C6) at the time of diagnosis (**a**), showing regression at 1 (**b**) and 2-year (**c**) follow-up, and stability after 3 (**d**) and 5 years (**e**). Other smaller lesions with similar features are evident in the D10-D11 (**a**) region that were less evident in the last control

An 8-year old child with two older brothers presented with global developmental delays, genu and cubitus valgum, and bilateral clubfoot with a secondary gait disturbance. He was diagnosed with NF1, presenting with more than six café-au-lait spots (> 5 mm), inguinal and axillary freckling, one large hairy patch on the hand and a vascular malformation on the right leg. The neurological exam was negative. He underwent an X-ray exam of the spine, because of a mild scoliosis, showing scalloping of the posterior wall of T6-T9 vertebral bodies for which we performed an MR exam of the spine. The MR scan showed three intramedullary hyperintense lesions on T2WI localized in the cervical and thoracic tracts. The largest of these lesions was localized in the cervical tract (C1-C6) and presented a tumor-like appearance and was initially diagnosed as a low-grade glial neoplasm. Since the patient didn’t present any new symptoms at the time of the exam, a wait-and-see approach was chosen. Three months later, a follow-up MR exam of the spine was performed, showing partial spontaneous regression of the cervical spine lesion and a stability of the two other T2WI-hyperintense spinal lesions. On the subsequent MR follow-up exams done annually for three years and then biannually for 6 more years, the cervical lesion showed further spontaneous regression and then was unchanged through the end of the studies (Fig. 1). All of the MR exams included post-contrast T1WI, and none of the lesions ever showed enhancement. In the light of these observations and evidence of clinical stability, the initial hypothesis of a low-grade glial neoplasm was abandoned and the lesions were reclassified as medullary UBOs.

### Case 2

This patient was a 12-year-old female with normal development and growth. She was the only child of a healthy couple. At the age of five, she presented with dysphagia, ataxia, typical cafè-au-lait spots and freckles, thus a clinical diagnosis of NF1 was made. The subsequent brain MR exam showed multiple cerebral and brainstem T2WI-hyperintense, non-enhancing lesions, which were diagnosed as UBOs, a diffuse fornix thickening, brainstem enlargement and a unilateral non-enhancing optic nerve glioma. An additional nodular lesion, which presented enhancement on post-contrast T1WI, was found in the right cerebellar hemisphere and was identified as a pilocytic astrocytoma. Additional exams, including ophthalmological and endocrinological essays revealed no further abnormalities.

The posterior fossa neoplasm showed clinical and radiological progression at further examinations and the patient underwent surgical removal of the lesion at the age of nine, with an improvement of the dysphagia and ataxia. She then developed a painful dorsal scoliosis for which X-rays of the spine were performed showing only a schisis of the posterior arch of S1, leading to a spinal MR to exclude the presence of neurofibromas. We found multiple small intramedullary lesions which were hyperintense on T2WI, had blurred margins, located mostly in the central medullary region and in the cervical spine, some of which presented a tumor-like appearance. None of these lesions showed enhancement on post-contrast T1WI. After a two-year MR follow-up, all of the lesions remained unchanged and were diagnosed as medullary UBOs.

### Case 3

A 28-year-old female with normal development and growth presented for care. At the age of twelve she was diagnosed with NF1, inherited from her father, and was noted to have lumbar scoliosis associated with lower limb length asymmetry, Lisch nodules, multiple cutaneous neurofibromas, cutaneous angiomas and freckles. The neurological exam was negative. A brain MR examination revealed bilateral pallidal UBOs.

**Fig. 2:**
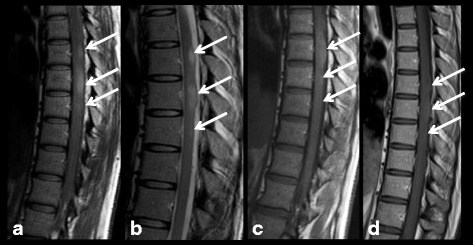
Patient number 3. Sagittal Turbo Spin-Echo PD-w (**a**), T2-w (**b**) and Turbo Spin-Echo T1-w, before (**c**) and after (**d**) i.v. contrast injection, sequences: 3 tumor-like dorsal spinal lesions which show high signal on PD, T1 and T2-w images without enhancement on post-contrast T1w sequences

She developed a painful dorsal scoliosis for which she underwent a spinal MR exam showing three T2WI and T1WI-hyperintense spinal lesions localized in the thoracic spine with a tumor-like appearance. None of these lesions showed enhancement on post-contrast T1WI. During the 9-year MR follow up, these lesions remained stable without the onset of any new neurological symptoms. Therefore, they were identified as medullary UBOs (Fig. 2).

### Case 4

**Fig. 3:**
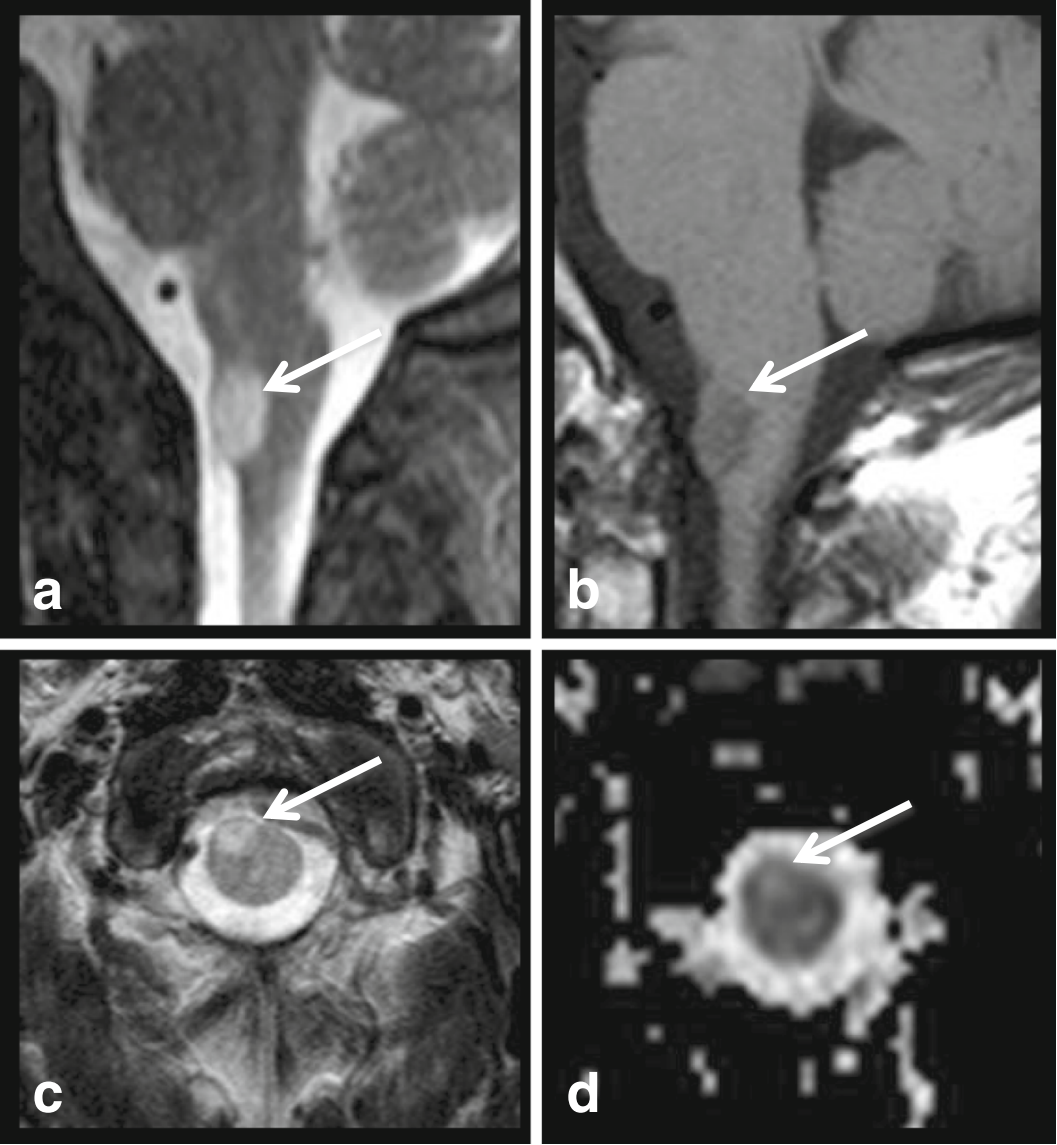
Patient number 4. Sagittal Turbo Spin-Echo T2-w (**a**), Spin-Echo T1-w (**b**), axial Turbo Spin-Echo T2-w (**c**) and axial ADC map (**d**). A well-defined, T2-hyperintense, T1-hypointense tumor-like lesion localized on the right side of the bulbo-medullary junction. It presents high signal intensity on the ADC map due to intramyelinic edema

A male patient who had a positive family history for NF1 (father) was evaluated. He was 14 years old at the time of the first exam in our institution. He presented with cutaneous neurofibromas, macrocephaly, and short stature. In addition, a bilateral optic nerve pallor was noted for which he underwent a brain MR, showing multiple UBOs, non-enhancing bilateral optic gliomas and a pilocytic astrocytoma of the left thalamus. The exam also showed a small T2WI-hyperintense spinal lesion at the level of C1. The subsequent spinal MR examination confirmed the isolated cervical tumor-like lesion on the right side of the cord which showed no enhancement after gadolinium injection and with well-defined margins (Fig. 3). This lesion also had high signal intensity in ADC maps. Follow up exams in the next two years showed no significant variation in the characteristics of the aforementioned lesion.

### Case 5

An 8-year-old male with no family history of NF1 was evaluated and found to have normal psychomotor development and growth. He presented typical features of NF1 and mild back pain for which a spinal MR exam was performed. It showed four small tumor-like lesions of the spinal cord which were moderately hyperintense on T2WI. These lesions were barely visible in sagittal scans, but axial images better defined the presence and characteristics of the aforementioned lesions. None of these lesions showed enhancement on post-contrast T1WI and they showed no evolution in the follow up exam one year later.

### Case 6

This 11-year-old male patient had no family history for NF1 but had global development delays and mild congenital hypotonia. At the age of two months he was diagnosed with NF1, presenting with typical cafè-au-lait spots, macrocephaly and freckles. A brain MR examination, performed at the age of 10, showed a pre-rolandic pilocytic astrocytoma for which the patient underwent surgery.

A spinal MR exam was performed because of the onset of a painful dorsal scoliosis. It showed four tumor-like lesions of the spinal cord and of the conus medullaris which were hyperintense on T2WI and showed no enhancement on post-contrast T1WI. A follow up exam after 6 months showed no variations. The neurological examination did not reveal any focal deficit.

### Case 7

A 7 year-old male patient was diagnosed at the age of 18 months with NF1, presenting typical cafè-au-lait spots and freckles, without a family history for this pathology. Neuropsycological development and growth were normal. He underwent the first MR exam at our institution as he presented persistent headache and a reduction of the visual acuity, showing a low-grade astrocytoma localized in the tegmentum of the midbrain causing a triventricular hydrocephalus. An incidental lesion of the cervical spine was detected for which a spine MR was performed. Three tumor-like, T2WI-hyperintense lesions were detected, none of which showed enhancement on T1WI after gadolinium injection. All of these were stable at follow-up MR exams after 12 months.

## Discussion

In previous studies, brain UBOs have been variously defined: hamartomas, altered myelination, or heterotopias [8–10]. In the only histological analysis of these lesions, vacuoles (5–100 μm) have been documented in the myelin sheath. This was correlated to intramyelinic edema. A reduction in the cellularity of the white matter was seen as well, together with an increased proliferation of glial cells [11].

More recent studies have confirmed this finding by using non-invasive MR techniques which showed a modification of the microstructural compartmentalization with an increase of extracellular-like intracellular water, an indication of intramyelinic edema, in UBOs confirming the previously mentioned histological findings [12].

In NF1, intramedullary lesions are more frequently low-grade astrocytomas (15% of patients) [13]. Rare cases of ependymomas (4 cases) [14] and gangliogliomas [15, 16] have also been reported in NF1 patients. These lesions are almost always symptomatic, single, have an inhomogeneous signal, often enhance in post-contrast T1WI and usually show progression in follow-up exams.

To our knowledge only one previous report of a medullary lesion in a patient with NF1, analogous to those herein documented in our patients, has been published as of this date. The lesion, in line with the knowledge of the time, was defined as a hamartomatous spinal cord lesion [17].

We report seven additional cases presenting such lesions localized in different segments of the spine, mainly in the cervical segment, describing their MR features and long-term clinical and radiological follow up. In our experience, patients more frequently had multiple asymptomatic medullary lesions, with a tumor-like appearance, always hyperintense in T2WI, hypo, iso or slightly hyperintense in T1WI, and were never enhancing in post-contrast T1WI. At follow up exams they were stable, except for one patient in which a spontaneous partial regression was documented (Table 1). For these characteristics, analogous to those of brain UBOs in NF1 patients, we have classified the spinal lesions of our patients as medullary UBOs (mUBOs).

**Table 1:** Summary of the patients’ anagraphical data and neuroradiological features

	Age	Sex	T1WI	T2WI	CE	Location (n°)	Follow-up (yrs)
Case 1	8	M	Hyperintense	Hyperintense	None	cervical (1), dorsal (2)	Regression (9)
Case 2	12	F	Isointense	Hyperintense	None	cervical (3), dorsal (2)	Stable (2)
Case 3	23	F	Hyperintense	Hyperintense	None	dorsal (3)	Stable (9)
Case 4	14	M	Hypointense	Hyperintense	None	cervical (1)	Stable (2)
Case 5	8	M	Isointense	Hyperintense	None	cervical (2), dorsal (2)	Stable (1)
Case 6	11	M	Isointense	Hyperintense	None	cervical (2), lumbar (2)	Stable (0,5)
Case 7	7	M	Isointense	Hyperintense	None	cervical (3)	Stable (1)

*M* male, *F* female, *CE* contrast enhancement, *n*° number, *yrs.* years

The limitations of our study are the small number of patients and the absence of a histological confirmation of our diagnosis due to the benign nature of the lesions which didn’t justify a biopsy especially in relation to their medullary localization. For these reasons we hope to expand the population for future studies. Even if screening for mUBOs is not an indication for routine spinal MR exams, MR studies of the spine may be done in case of other symptomatic lesions. Knowledge about these benign mUBOs is critical for the correct interpretation of the images and providing appropriate prognostic information.

## Conclusions

The presence of mUBOs might have been severely underestimated in the past due to the asymptomatic nature of these lesions. In all our patients the diagnosis was made incidentally in spinal or brain MR exams they underwent for concurrent symptomatic conditions (painful scoliosis, brain lesions). The aim of our work is to increase awareness of the possibility of mUBOs in NF1 patients which can simulate neoplastic lesions, suggesting a more conservative approach.

## Abbreviations

CNS: Central nervous system
MR: Magnetic resonance
mUBOs: Medullary unidentified bright objects
NF1: Neurofibromatosis type 1
NIH: National Institute of health
T1WI: T1-weighted images
T2WI: T2-weighted images
UBOs: Unidentified bright objects
